# Biocompatibility Analyses of Al_2_O_3_-Treated Titanium Plates Tested with Osteocyte and Fibroblast Cell Lines

**DOI:** 10.3390/biomedicines5020032

**Published:** 2017-06-16

**Authors:** Alberto Smargiassi, Jessika Bertacchini, Marta Checchi, Francesco Cavani, Marzia Ferretti, Carla Palumbo

**Affiliations:** Dipartimento di Scienze Biomediche, Metaboliche e Neuroscienze, Sezione di Morfologia Umana, Università di Modena e R.E, 41124 Modena, Italy; jessika.bertacchini@unimore.it (J.B.); marta.checchi@unimore.it (M.C.); francesco.cavani@unimore.it (F.C.); marzia.ferretti@unimo.it (M.F.); carla.palumbo@unimore.it (C.P.)

**Keywords:** osseointegration, titanium plate, osteocyte, biocompatibility, implants

## Abstract

Osseointegration of a titanium implant is still an issue in dental/orthopedic implants durable over time. The good integration of these implants is mainly due to their surface and topography. We obtained an innovative titanium surface by shooting different-in-size particles of Al_2_O_3_ against the titanium scaffolds which seems to be ideal for bone integration. To corroborate that, we used two different cell lines: MLO-Y4 (murine osteocytes) and 293 (human fibroblasts) and tested the titanium scaffolds untreated and treated (i.e., Al_2_O_3_ shot-peened titanium surfaces). Distribution, density, and expression of adhesion molecules (fibronectin and vitronectin) were evaluated under scanning electron microscope (SEM) and confocal microscope (CM). DAPI and fluorochrome-conjugated antibodies were used to highlight nuclei, fibronectin, and vitronectin, under CM; cell distribution was analyzed after gold-palladium sputtering of samples by SEM. The engineered biomaterial surfaces showed under SEM irregular morphology displaying variously-shaped spicules. Both SEM and CM observations showed better outcome in terms of cell adhesion and distribution in treated titanium surfaces with respect to the untreated ones. The results obtained clearly showed that this kind of surface-treated titanium, used to manufacture devices for dental implantology: (i) is very suitable for cell colonization, essential prerequisite for the best osseointegration, and (ii) represents an excellent solution for the development of further engineered implants with the target to obtain recovery of stable dental function over time.

## 1. Introduction

Titanium is the metal of choice for prosthetics, internal fixation, and inner body devices but more importantly is a widely-used material for dental implants [1]. Introduced into surgery in the 1950s, titanium is still the most used metal in dentistry and since then several studies to improve the bio-interactions and adhesion in human body have been carried on [2,3]. The biocompatibility is due to its resistance to corrosion from body fluids, bio-inertness, high fatigue limit, and its capacity for osseointegration [4].

Osseointegration is a long and complicated process which had shown many problems in implants durable over time: biological rejection of the implant, incorrect or weak in-site adhesion or hypersensitivity reactions to implants in orthopedic and trauma surgery [5,6,7,8].

The quality of osseointegration in titanium implants is primarily due to: osteoblast differentiation into osteocyte that show and maintain its characteristic dendritic shape; stimulation of adhesion factors, specifically issued by bone cells; formation of cellular interconnections which allow cell communication and, in turn, easier proliferation and differentiation [9,10,11,12,13,14]. Particular attention has been given in recent decades to the morphology of the surface of titanium implants to overcome the biocompatibility issues [2,15,16]. The importance of the titanium morphology in the proliferation and differentiation of cells in vitro was showed by various authors. Karoussis et al. [17] demonstrated that surface morphology influences the proliferation and differentiation potential of MG-63 osteoblast-like cells; Brugge et al. [18] observed that titanium roughness can affect the initial interaction of cells (human osteosarcoma U2OS cells) with the material. Different methods of treatment of the surfaces are currently used to improve their biocompatibility, especially the two most commonly used: physical and chemical treatments. The most common methods of surface treatment currently used to improve the material biocompatibility are those implying physical and chemical agents. In particular, chemical treatments, such as graphene oxide, enhance the bone formation by increasing osteoblastic MC3T3-E1 cell proliferation rate; while, the surfaces coating with Poly(ε-caprolactone) and Poly(propylene fumarate) ameliorate the adhesion capability of cultured cells [19,20,21,22]. Moreover, physical treatment such as hydroxyapatite deposition or TiO_2_/silicate coating on titanium plates significantly influences the hydrophilicity, protein adsorption, and in vitro bioactivity of biomaterials [23,24]. Some authors suggested that sand-blasting treatment is an effective method to greatly improve the surface bio-performances of implant biomaterials [25]. Smeets et al. [26] showed that dental implants were successfully used for a number of years focusing on sandblasting, acid-etching, and hydrophilic surface textures; hereafter, new techniques like discrete crystalline deposition (DCD), laser ablation, and surface coatings with proteins, drugs, or growth factors were proposed [26]. All together these data suggest that these types of surface modification enhance the osseointegration. The pro and cons of the various methods are reported in literature [2,16]; in the present article, we will take a closer look of one peculiar and innovative method of physical surface treatment used to create a more suitable environment for bone. For this reason, it is crucial to study and develop an osseo-compatible and long-time resistant surface in titanium implants.

Hence, for the present study, engineered biomaterials (i.e., shot-peened titanium surfaces) were obtained by shooting different-in-size particles of Al_2_O_3_ against the scaffolds of biomaterial. Then, two different cell lines, MLO-Y4 (murine osteocytes) and 293 (human fibroblasts), were cultured on untreated and treated surface of titanium biomaterials. The choice of such cell lines was made on the basis of what was already demonstrated during the occurrence of the implant osseointegration: after the removal of the inflammatory tissue and issuing of cytokines, fibroblasts secrete the preliminary type-1 collagen texture under which osteoblast-transforming-osteocytes start to build new woven bone, which is then consolidated by lamellar bone, inhabited by osteocytes considered the bone mechanosensors [13] and responsible, with their signaling, to trigger and maintain osseointegration stable over time. It is therefore important that just those two cell types (fibroblasts and osteocytes) work on a suitable surface, whose characteristic play a crucial role. Distribution, density, and expression of adhesion molecules (fibronectin and vitronectin) were analyzed with confocal (CM) as well as scanning electron microscopy (SEM) as previously described [27] in order to evaluate the suitability of the treated biomaterial for cell colonization and its possible use in dental and bone implants.

## 2. Materials and Methods

### 2.1. Titanium Plate Preparation and Surface Analysis

Sixteen titanium plates were furnished by Safe & Simple (Treviso, Italy); the surfaces of half of them were treated by shooting different-in-size particles of Al_2_O_3_ against the titanium following the provided methods by Safe&Simple. Aluminum oxide particles (White Corundum) with irregular profile (granules), different from the classical spherical particles used in other surface treatments [27], were projected on the titanium surface by compressed air machine (gun pressure: 1.5 bar, gun nozzle diameter: 10mm, nozzle-surface distance: 100 mm, impact angle: 90°, roughness obtained: *R*a = 1.0–1.2). All the samples were then cleaned in absolute alcohol and sterilized following a standard procedure in an autoclave. Before cell culturing, titanium plates were analyzed under a scanning electron microscope in order to appreciate the difference after the surface treatment.

### 2.2. Cell Line Culture and Sample Preparation

Murine long bone osteocyte Y4 (MLO-Y4) cell line was used in order to test the osteocyte-like cell adhesion, distribution, viability, and growth on titanium plates. Briefly, the cells were cultured on collagen-coated plastic petri dish and grown at 37 °C, 5% CO_2_, 95% air using DMEM containing ribonucleosides, deoxyribonucleosides, and l-glutamine, supplemented with 5% fetal bovine serum (FBS), 5% bovine calf serum (CS) and penicillin/streptomycin at 100 U/mL. Cells were initially defrosted and plated at a concentration of 150,000 cells/well, then sub-cultured once they reached the 80% of confluence.

Fibroblastic cell line 293 was also used to test the cell adhesion, distribution, viability, and growth on titanium plates. Cells were cultured on 75 cm^2^ flasks at 37 °C, 5% CO_2_, 95% air at an initial concentration of 10^4^/cm^2^ using DMEM containing ribonucleosides, deoxyribonucleosides, and l-glutamine, supplemented with 10% fetal bovine serum (FBS) and penicillin/streptomycin at 100 U/mL. Cells were sub-cultured once they reached the 90% of confluence.

In order to prepare the samples for investigations, all the titanium plates were placed into multi-wells plates and covered with standard media (DMEM supplemented with 10% FBS and penicillin/streptomycin); four treated plates were cultured with MLO-Y4 cell line and four treated plates were cultured with 293 cell line; four untreated plates were cultured with MLO-Y4 cell line and four untreated plates were cultured with 293 cell line. All plates were cultured with an initial cell concentration of 10^4^ cell/well for 48 h. Samples were then fixed in paraformaldehyde 4% in PBS (pH 7.4) for 20 min.

### 2.3. Confocal Microscopy (CM)

All fixed samples were rinsed in phosphate buffer and then permeabilized with 0.1% Triton X-100 in PBS. Samples were covered with PBS containing 3% Bovin serum albumin (BSA) for 30 min at room temperature (RT), then permeabilized samples were incubated for 1 h at RT with primary antibodies (mouse anti-Vitronectin and rabbit anti-Fibronectin), diluted 1:200 in PBS containing 3% BSA. After washing, samples were incubated for 1 h at RT with secondary antibodies (Cy5-conjugated donkey anti-rabbit; FITC-conjugated sheep anti-mouse), diluted 1:200 in PBS containing 3% BSA.

Confocal fluorescence analysis was performed using a Leica TCS SP2 AOBS (Leica Microsystems, Wetzlar, Germany) confocal laser-scanning microscope. Confocal images were processed with Leica LCS soft-ware (version Lite 2.61.1537, Leica Microsystem, Wetzlar, Germany) for investigations.

### 2.4. Scanning Electron Microscopy (SEM) and X-ray Microanalysis

Treated and untreated titanium plates, plated with both MLO-Y4 and 293 cell lines, were observed with the ESEM Quanta-200 scanning electron microscope (Fei Company, Hillsboro, OR, USA) under low vacuum condition and in backscattered mode after gold-palladium sputtering of samples. Samples’ compositions were also analyzed via X-ray microprobe using the ESEM software (version 4.07, Oxford Instruments Analytical, Tubney newlyWoods, Abingdon, Oxon, UK).

## 3. Results

### 3.1. Surface Morphology

**Figure 1:**
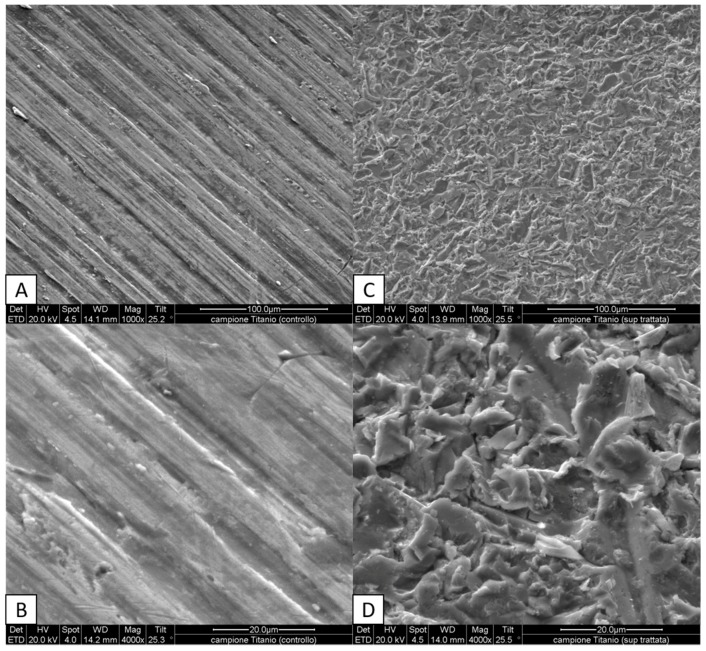
Morphology of titanium surfaces analyzed under scanning electron microscope (SEM): untreated surfaces (**A**,**B**) may be easily distinguished from treated surfaces (**C**,**D**) where variously-shaped spicules are appreciable. Magnifications and scales are reported in figure (**A** and **C** 1000×; **B** and **D** 4000×).

The first analysis was aimed to observe the modified surface of the treated titanium plates. The treated engineered biomaterial surfaces, observed under SEM, showed an irregular morphology due to variously-shaped spicules, unlike the untreated ones which showed a regularly striated appearance (Figure 1). Linear stripes are appreciable on the untreated surfaces of titanium plates (Figure 1 panels A and B). This shape may be attributed to the cutting method used to create these plates. These first observations demonstrate the effectiveness of the sample preparation and the differences between the two scaffolds.

### 3.2. Cell Viability, Distribution, and Adhesion Factor Expression: MLO-Y4 vs. 293 in Treated vs. Untreated Titanium Plates

**Figure 2:**
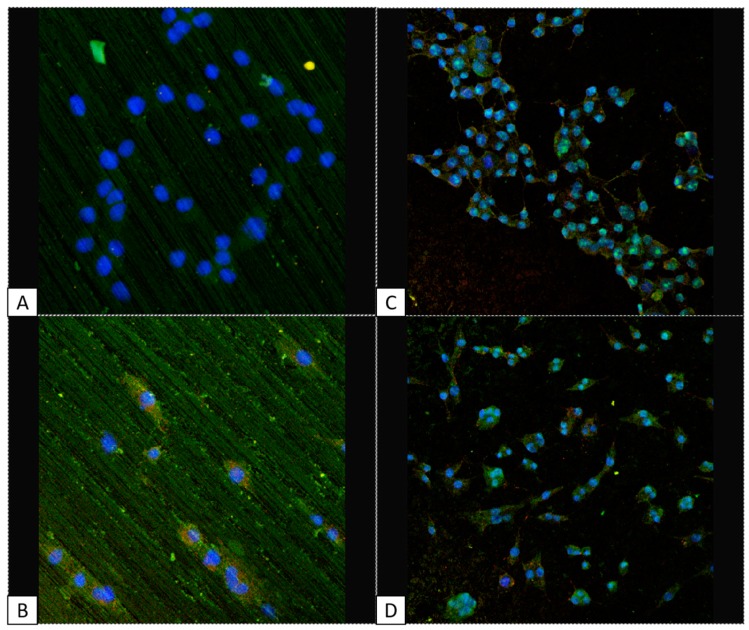
Confocal microscope images: treated titanium surfaces seems to be more suitable for cell growth and adhesion (panels **C**,**D**) compared with untreated one (panels **A**,**B**) for both cell lines: MLO-Y4 (panels **B**–**D**) and 293 (panels **A**–**C**). DAPI in figure marks nuclei in blue, FITC marks vitronectin in green, TRITC marks fibronectin in red. Panel A and B: 2500×, panel C and D: 1000×.

Treated and untreated titanium scaffolds were cultured with MLO-Y4 and 293 cell lines in order to analyze the cell distribution and viability as well as the expression of two adhesion factors, Vitronectin and Fibronectin. Dapi was used to mark the nuclei, meanwhile Fitc and Tritc markers were used to track vitronectin and fibronectin, respectively. As expected, the cell viability was much higher in the treated titanium plates compared with the untreated ones, for both the cell lines, demonstrating the better suitability of the treated versus untreated titanium plate for biological implants (Figure 2). In the same image, the homogenous cell distribution of both cell lines in the treated titanium plates (Figure 2C,D) is also appreciable, meanwhile in the untreated titanium plates the cells appear arranged in rows (Figure 2A,B). The expression of vitronectin and fibronectin were easily detected in both cell lines when grown on the treated titanium plates, meanwhile almost no signal was detected for growth on untreated plates (Figure 2).

### 3.3. Cell Shape: MLO-Y4 under CM

**Figure 3:**
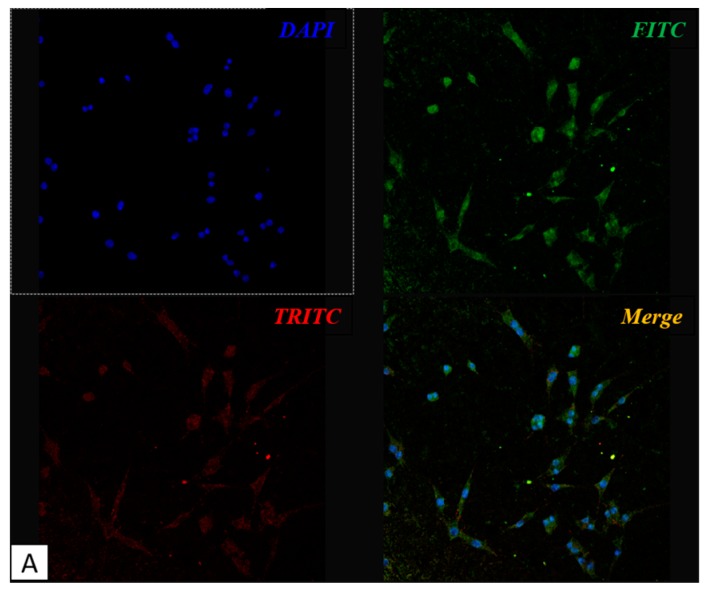

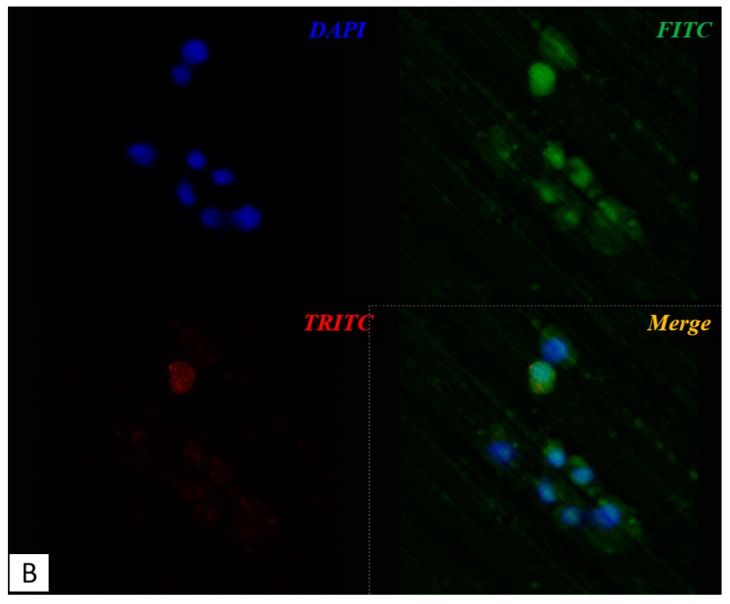
Confocal microscope images: MLO-Y4 cell growth on treated plates (**A**) shows the dendritic shape characteristic of osteocyte, meanwhile cells maintain a rounder shape when grown on untreated surface (**B**). DAPI in figure marks nuclei in blue, FITC marks vitronectin in green, TRITC marks fibronectin in red. Panel A: 1000×, panel B: 4000×.

As previously mentioned, the maintenance of osteocyte dendritic shape is a fundamental factor to determine the quality of osseointegration. We then focused our attention on MLO-Y4 and the presence of the characteristic dendritic shape which is typical of healthily differentiated osteocytes. We marked the cells with Fitc and Tritc to track the distribution of vitronectin and fibronectin on the two kinds of titanium plates. As already observed (Figure 2), both adhesion molecules are much less expressed in untreated titanium plates compared to treated ones, strengthening our hypothesis that treated titanium plates are more suitable for cell adhesion. Moreover, it is evident the typical osteocyte dendritic shape in MLO-Y4 cell line when grown on treated titanium plates; when seeded on untreated titanium surface, the same cells are less numerous and show a more round shape (Figure 3).

### 3.4. Cell Shape: MLO-Y4 under SEM

**Figure 4:**
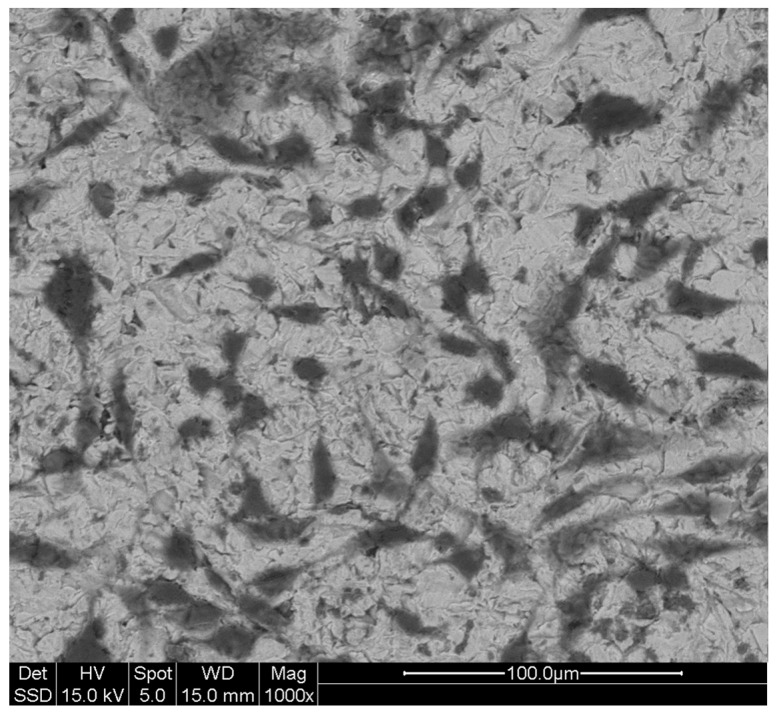
MLO-Y4 under scanning electron microscope (SEM): Characteristic dendritic shape of MLO-Y4 cultured on treated titanium plates is also appreciable under SEM analysis where cell bodies and cytoplasmic processes (dendrites) are easily identifiable thanks to the adjustable gray contrast resulting from the analysis after the gold-palladium sputtering of samples. Magnifications and scale are reported in the figure.

The dendritic shape of MLO-Y4 cell line grown on titanium plates was also analyzed under scanning electron microscope (SEM) to obtain more defined and appreciable details (Figure 4). The images obtained underline even more the well-maintained cell-shape of MLO-Y4 with the typical dendritic aspect indicating good cell viability.

**Figure 5:**
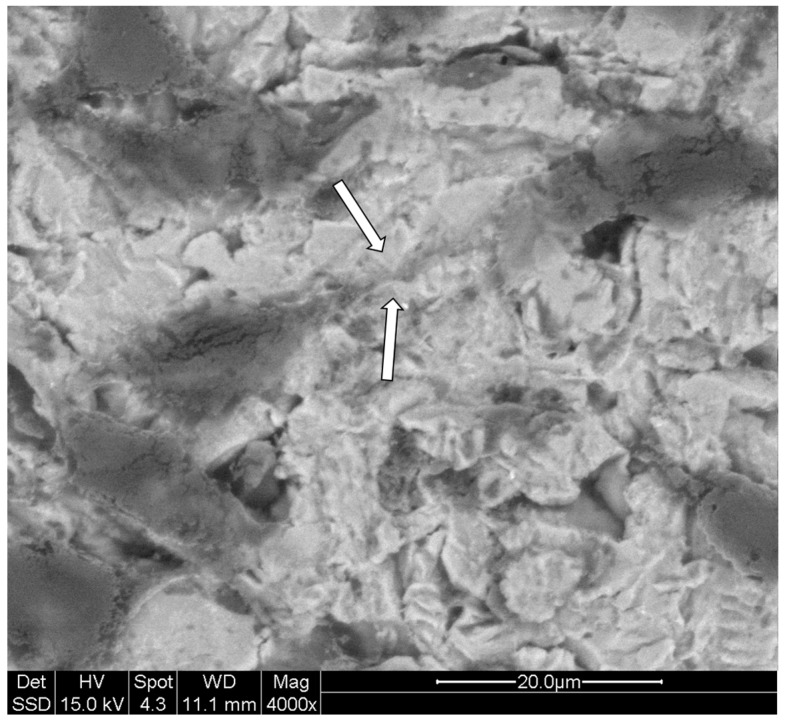
MLO-Y4 under scanning electron microscope (SEM): Dendrites of MLO-Y4 cells are spotted widely on the surfaces of treated titanium plates. In the figure, at higher magnification, two cell dendrites connecting each other (white arrows) can be appreciated. Magnification and scale are reported in figure.

Besides the osteocyte-like shape, equally important is the presence of cell connections in those cells grown on titanium plates. In SEM analysis, it is possible to appreciate, under higher magnifications, the presence of cellular contacts at the extremities of dendrites of the cells plated (Figure 5). As previously mentioned, the presence of these junctions is fundamental for cell communications, cell survival, growth and differentiation. It is then of extreme importance that these cells not only present a dendritic shape but also that the cytoplasmic processes may find a way to connect to each other as shown by our results (Figure 4 and Figure 5).

### 3.5. X-ray Microanalyses: MLO-Y4

**Figure 6:**
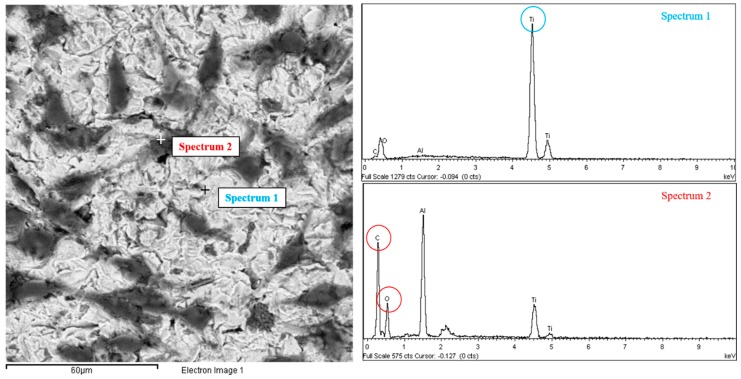
MLO-Y4 under scanning electron microscope (SEM) with X-ray analysis: Two points of a treated titanium plate cultured with MLO-Y4 cell line are analyzed with the X-ray analysis of the SEM resulting in two spectra of components. In the SEM image, the darker gray area indicated as “Spectrum 2” is confirmed to be of organic nature, due to the high peaks of Oxigen (O) and Carbon (C), meanwhile the absence of those peaks and the prevalence of Titanium peak (Ti) confirm the inorganic nature of the lighter gray area indicated as “Spectrum 1”.

SEM analysis of MLO-Y4 was completed by X-ray microanalyses conducted on the surfaces of the treated titanium plates in order to confirm the validity of our results. Through the analysis software of the X-ray machine integrated in the scanning electron microscope was possible to evaluate the presence of every element present on the surface of the titanium plates. X-ray microanalysis was performed in different places of the treated titanium plates, on both the naked surface of the titanium plate and the cell surface to confirm, through the detection of carbon (C) and oxygen (O), the biological presence of cell protoplasm and exclude artifacts. The two spectrua obtained clearly show how only titanium (Ti) peaks were present on the brighter area indicating the uncovered biomaterial, whereas the carbon (C) and oxygen (O) peaks were present on the darker area indicating the organic nature of the cells covering the biomaterial surface (Figure 6).

## 4. Discussion

Titanium is, to date, the most common biomaterial used in dental implants. Many different industrial treatments are currently used to modify the implant surface in order to obtain an optimal surface roughness and topography. In our study, we evaluate the biocompatibility of titanium plates that received a new surface treatment method (i.e., granules instead of spherical particles shooting) with the aim to improve cell adhesion and viability. Adhesion and viability of osteocytes on biomaterials is crucial for an implantation durable over time in bones. Tests with cellular models have been largely used to predict the fate of implantations with different treated surfaces and allow to develop new and more suitable processes of treatment of titanium and other biomaterials. The MLO-Y4 cell line has been largely used to mimic the behavior of osteocytes in in vitro experiments. In the present study, we evaluate the quality of the biocompatibility of a new type of treatment of titanium surfaces in an in vitro model, using fibroblastic and osteocyte cell lines, involved in triggering and maintaining the osseointegration, respectively. The surfaces of treated and untreated titanium plates were compared with and without the cell lines. The process used on the surfaces of treated plates created an irregular shape characterized by variously-shaped spicules compared to the smooth untreated one. Irregular surfaces have been largely reported to be more suitable for biocompatibility purposes, in fact along with the use of different materials to ameliorate implant surface, several studies evaluated physical characteristics that can improve both the regeneration and integration of bone tissue e.g., titanium oxidation [28], electrical stimulation of surfaces [29], and different sterilization methods [30]. Moreover, studies on cells cultured in vitro on titanium scaffolds characterized by differently-porous structures clearly demonstrated that microtopography and geometry effect can affect substantially the “cell-scaffold” interaction [31,32,33]. The difference in growth, cell distribution, and viability of both the cell lines in treated plates vs. untreated plates can be easily appreciated in the confocal-microscope and electron microscope images reported in the present paper. Moreover, the presence and intensity of fluorescence signals of vitronectin and fibronectin was found to be higher in the plated cells on the treated surfaces when compared with the untreated surfaces of titanium plates. This result underlines the difference in adhesion evidenced by the cell line when cultured on irregular shaped surfaces. In particular, the huge differences showed in our results highlight the quality of the new performed process to treat titanium surfaces used in our study. Even more important evidence of the quality of the treated surface for osteocyte adhesion (and, in turn, for osseointegration) is the formation of cellular dendrites and contacts among the plated MLO-Y4 cells. Cell shape is the main indicator of the health of bone cells; in particular the typical dendritic shape of osteocytes (that in our analyses was never observed on untreated plates) is an essential requisite for the maintenance of differentiation and functionality. Our confocal images clearly evidence how the treated surfaces of the titanium plates stimulate MLO-Y4 to grow maintaining the typical dendritic shape with long cytoplasmic processes which connect the cells to each other granting reciprocal communication, which is fundamental for bone health; in fact, well-developed intercellular relationships triggered by osteocyte dendrite-induced signals are necessary to perform balanced remodeling processes in maintaining the physiological bone turnover. These results are confirmed by our SEM analysis and any bias due to artifacts is wiped out by X-ray microanalysis which clearly shows the organic nature of the connected dendrites. The surface morphology (clearly appreciable in Figure 1, panels A and C) obtained by our treatment differs from others commercially available for the fact that it resembles much more (with respect to the classical ones) the irregular profile of bone surfaces recognized by osteogenic cells; such a feature is crucial for cell adhesion and bone deposition processes, an essential precondition for the best osseointegration.

## 5. Conclusions

In conclusion, standing the fact that the cell suitability of a biomaterial is one of the most complicated characteristics in the regeneration strategies, the results obtained showed that this type of Al_2_O_3_-treated titanium biomaterial is more suitable for cell colonization (essential prerequisite for triggering and maintaining over time a good osseointegration) than the untreated titanium plates and represents an excellent solution for the development of further engineered implants with the target to obtain recovery of dental and bone function stability over time.
